# Supplementary material for: DeepRePath: Identifying the Prognostic Features of Early-Stage Lung Adenocarcinoma Using Multi-Scale Pathology Images and Deep Convolutional Neural Networks
Source: Cancers (Basel). 2021 Jul 1;13(13):3308. doi: 10.3390/cancers13133308 (PMC8268823; doi:10.3390/cancers13133308)
Supplement: Supplementary file 1 [file cancers-13-03308-s001.zip › Revised Manuscript_cancers-1243624-supplementary- for decision/supplemenatry figure 1.pdf]

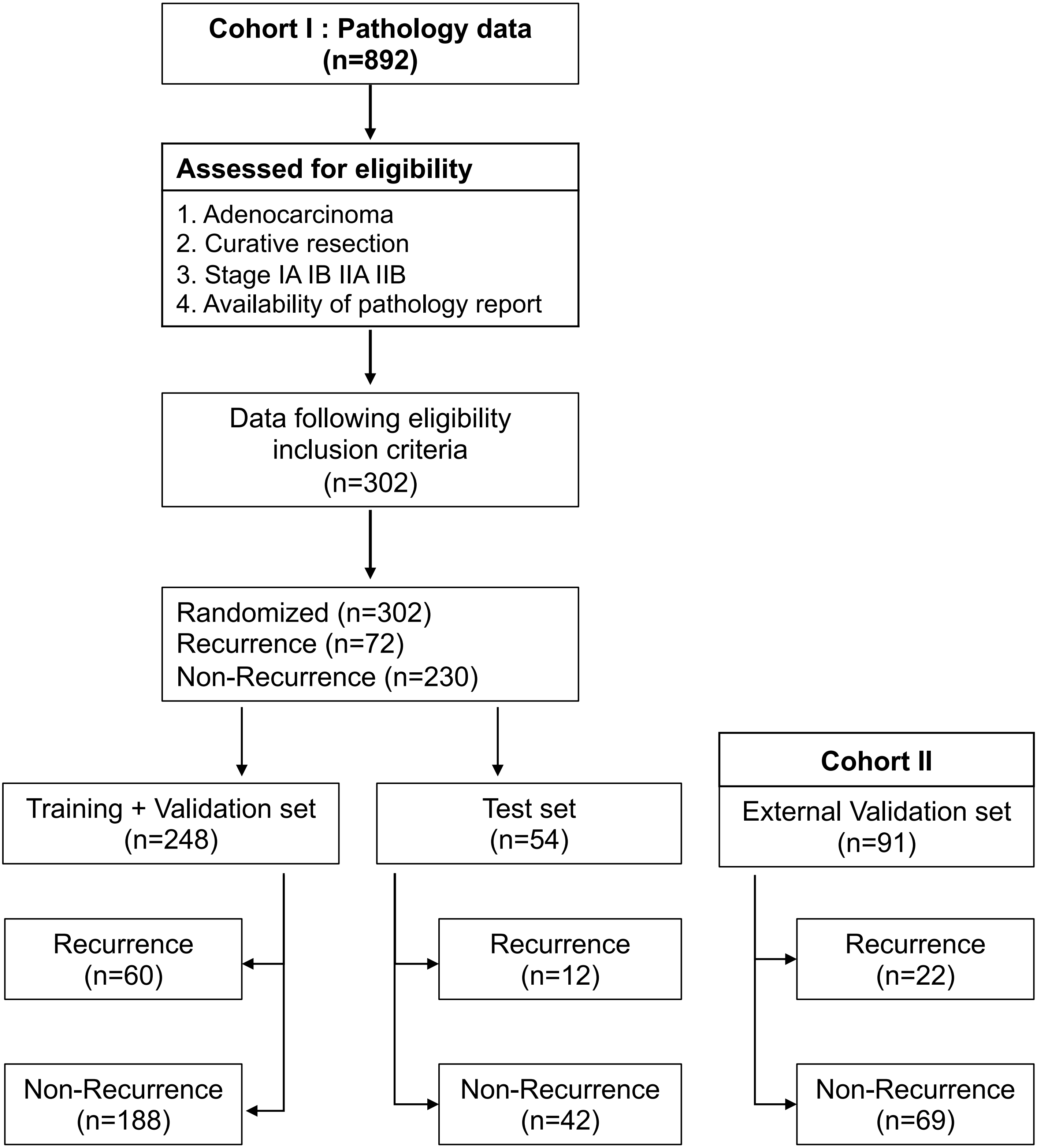

Cohort I : Pathology data
(n=892)
Assessed for eligibility
1. Adenocarcinoma
2. Curative resection
3. Stage IA IB IIA IIB
4. Availability of pathology report
Data following eligibility inclusion criteria
(n=302)
Randomized (n=302)
Recurrence (n=72)
Non-Recurrence (n=230)
Training + Validation set
(n=248)
Test set
(n=54)
Cohort II
External Validation set
(n=91)
Recurrence
(n=60)
Non-Recurrence
(n=188)
Recurrence
(n=12)
Non-Recurrence
(n=42)
Recurrence
(n=22)
Non-Recurrence
(n=69)
