# Supplementary material for: DeepRePath: Identifying the Prognostic Features of Early-Stage Lung Adenocarcinoma Using Multi-Scale Pathology Images and Deep Convolutional Neural Networks
Source: Cancers (Basel). 2021 Jul 1;13(13):3308. doi: 10.3390/cancers13133308 (PMC8268823; doi:10.3390/cancers13133308)
Supplement: Supplementary file 1 [file cancers-13-03308-s001.zip › Revised Manuscript_cancers-1243624-supplementary- for decision/supplementary figure 2.pdf]

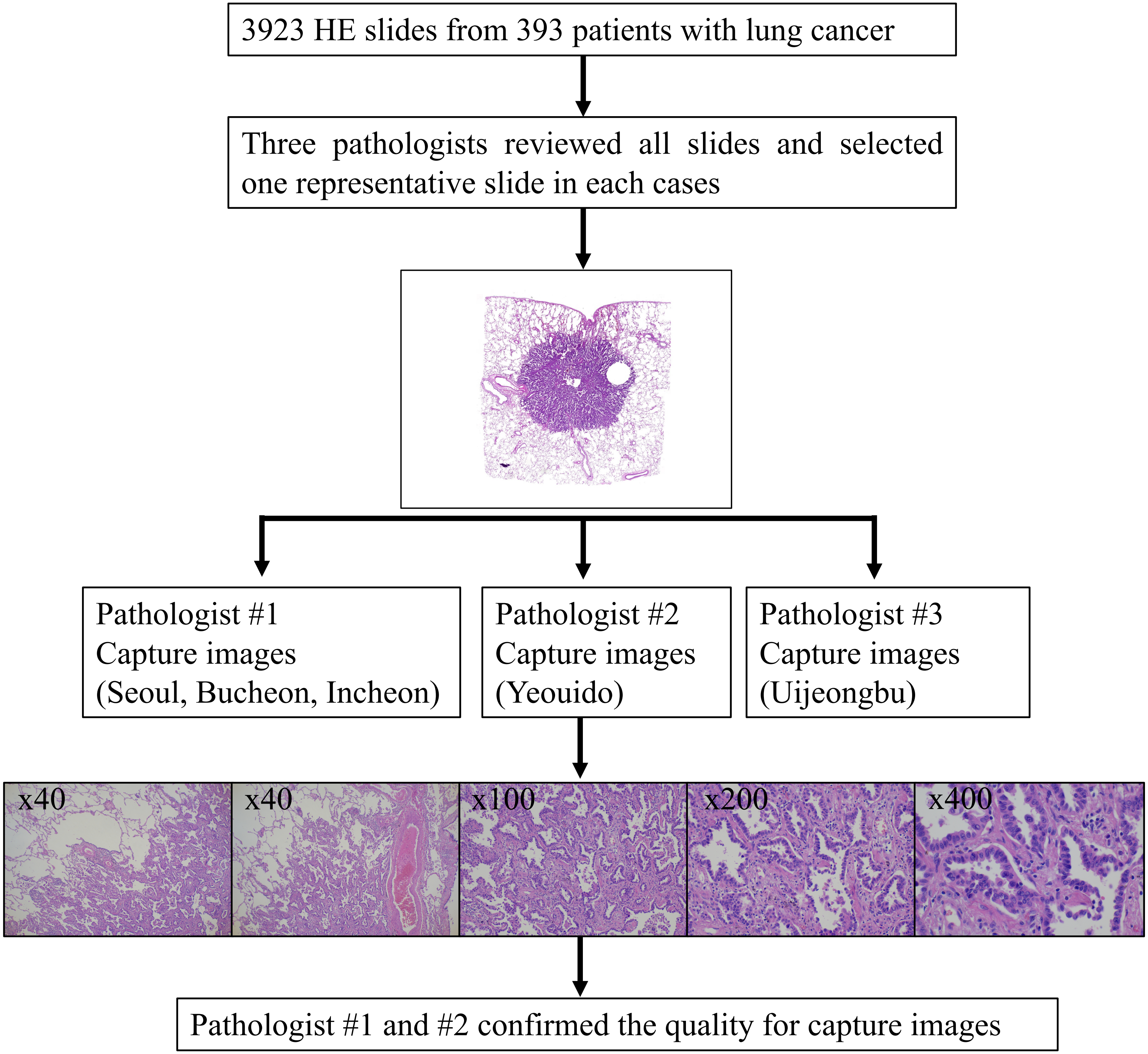
3923 HE slides from 393 patients with lung cancer
Three pathologists reviewed all slides and selected one representative slide in each cases
Pathologist #1
Capture images
(Seoul, Bucheon, Incheon)
Pathologist #2
Capture images
(Yeouido)
Pathologist #3
Capture images
(Uijeongbu)
x40
x40
x100
x200
x400
Pathologist #1 and #2 confirmed the quality for capture images
