# Supplementary material for: DeepRePath: Identifying the Prognostic Features of Early-Stage Lung Adenocarcinoma Using Multi-Scale Pathology Images and Deep Convolutional Neural Networks
Source: Cancers (Basel). 2021 Jul 1;13(13):3308. doi: 10.3390/cancers13133308 (PMC8268823; doi:10.3390/cancers13133308)
Supplement: Supplementary file 1 [file cancers-13-03308-s001.zip › Revised Manuscript_cancers-1243624-supplementary- for decision/supplementary tables_R3_F.pdf]

Supplementary Table 1. Baseline characteristics of the cohort I and cohort II (external validation set)

Characteristic		Total	Cohort I	Cohort II	
		n = 393	n = 302	n = 91	P*
Age (years)	Median (range)	64 (25–86)	64 (25–86)	63 (35–83)	0.435
Gender	Male	204	148	56	0.036
	Female	189	154	35	
Differentiation	WD (%)	132	94	38	0.077
	MD (%)	208	165	43	
	PD/UD (%)	53	43	10	
Tumor size	Median, cm (range)	2.3 (0.6–12)	2.4 (0.6–12)	2.2 (0.6–6.5)	0.166
No. of LN positivity		0 (0–10)	0 (0–10)	0 (0–6)	
T stage	T1	216	160	56	0.15
	≥ T2	177	142	35	
N stage	N0	348	267	81	0.875
	≥ N1	45	35	10	
TNM stage	I	315	239	76	0.359
	≥ II	78	63	15	
Lymphovascular invasion	Yes	126	109	17	0.002
Adjuvant treatment	Yes	92	69	23	0.344
Recurrence	Yes (< 3years)	94	72	22	0.948
	No (> 3years)	299	230	69	

LN, lymph node; WD, well-differentiated; MD, moderately differentiated; PD, poorly differentiated; UD, undifferentiated;

P*, A two-sided P-value of <0.05, was considered statistically significant. Categorical variables were compared using the chi-square test

Supplementary Table 2. The institutes of the cohort I and cohort II (external validation set)

**Cohort I**				
Institute	Training set (%)	Validation set (%)	Internal test set (%)	Total
Inceon	22 (9.9)	3 (12.0)	5 (9.3))	30
Uijeongbu	15 (6.7)	2 (8.0)	3 (5.5)	20
Seoul	186 (83.4)	20 (80.0)	46 (85.2)	252
Total	223	25	54	302
**Cohort II**				
Institute	External validation set (%)			
Yeouido	39 (42.9)			
Bucheon	52 (57.1)			
Total	91			

Supplementary Table 3. Comparison of the performance of models using XGBoost, Gradient Boosting, and AdaBoost for cohort I (training set) and cohort II (external validation set)

Cohort	Model	Sensitivity (%)	Specificity (%)	PPV (%)	NPV (%)	Accuracy (%)	AUC
I	DeepRePath with XGBoost	74	78	59	89	77	0.77
	DeepRePath with Gradient Boosting	71	71	49	87	71	0.73
	DeepRePath with AdaBoost	65	73	52	86	71	0.72
II	DeepRePath with XGBoost	86	74	51	94	77	0.76
	DeepRePath with Gradient Boosting	71	82	55	90	79	0.75
	DeepRePath with AdaBoost	78	53	33	89	59	0.54

XGBoost, eXtreme gradient boosting; AdaBoost, adaptive boosting; PPV, positive predictive value; NPV, negative predictive value; AUC, area under the curve; TL, transfer learning
